# Plasmonic plano-semi-cylindrical nanocavities with high-efficiency local-field confinement

**DOI:** 10.1038/srep40071

**Published:** 2017-01-11

**Authors:** Feifei Liu, Xinping Zhang, Xiaohui Fang

**Affiliations:** 1 Institute of Information Photonics Technology and College of Applied Sciences, Beijing University of Technology, Beijing 100124, P. R. China

## Abstract

Plasmonic nanocavity arrays were achieved by producing isolated silver semi-cylindrical nanoshells periodically on a continuous planar gold film. Hybridization between localized surface plasmon resonance (LSPR) in the Ag semi-cylindrical nanoshells (SCNS) and surface plasmon polaritons (SPP) in the gold film was observed as split bonding and anti-bonding resonance modes located at different spectral positions. This led to strong local field enhancement and confinement in the plano-concave nanocavites. Narrow-band optical extinction with an amplitude as high as 1.5 OD, corresponding to 97% reduction in the transmission, was achieved in the visible spectrum. The resonance spectra of this hybrid device can be extended from the visible to the near infrared by adjusting the structural parameters.

Localized surface plasmon resonance (LSPR) as a result of collective oscillation of free electrons in noble metals have been investigated extensively either in single nanostructures^1^,^2^,^3^,^4^ or in hybrid systems like dimmers, oligomers, core-shells, particle-film systems, and etc.^5^,^6^,^7^,^8^,^9^,^10^,^11^,^12^,^13^,^14^,^15^,^16^,^17^,^18^,^19^,^20^,^21^,^22^,^23^,^24^,^25^,^26^,^27^,^28^. These hybrid systems generally involve multipolar plasmons with enhanced or newly generated coupling processes. Plasmonic hybridization may also enable improved tunability through adjusting the physical parameters, such as the size and/or the separation distance between the nanostructures.

A typical hybrid design consisting of metallic nanoparticles and a metallic film with a dielectric spacer, which is known as the metal-insulator-metal (MIM) system, has been extensively investigated^5^,^6^,^7^,^8^,^9^,^10^,^11^,^12^,^13^,^14^,^15^,^16^,^17^,^18^,^19^,^20^,^21^. The near-field interaction between particle plasmons and delocalized surface plasmons confines huge optical energy in the dielectric spacer and subsequently induces the out-of-plane plasmonic nanocavity resonance. Such a resonance mode depends strongly on the thickness of the spacer, where strong confinement was observed for a spacer thickness smaller than 50 nm^5^,^6^,^7^,^8^,^9^,^10^,^11^,^12^,^13^,^14^,^15^,^16^,^17^. With increasing the spacer thickness, the confinement becomes weaker and part of the hybrid resonance mode radiates out of the system, decreasing spectral selectivity with broadened line width^18^,^19^,^20^,^21^. Increasing the size of the nanoparticles not only enhances the plasmonic local field and nanocavity confinement, but also reduces the sensitivity of the hybrid resonance mode to the spacer thickness. Thus, higher-efficiency hybrid plasmonic resonance in the infrared has been reported^18^,^19^. Furthermore, such a MIM system has been incorporated into the bow-tie nanoantennas and much enhanced local field may be achieved in the extremely small gap^20^. Bonding and antibonding plasmonic resonance modes are also typical features of the hybrid systems^22^,^23^,^24^,^25^,^26^,^27^,^28^, which have been observed in metallic nanoshells provided by nanorings^22^,^23^, nanocups^24^, and the silica-gold core-shell nanoparticles^25^.

In this work, we achieved high-efficiency notch filtering effect in the visible spectral range through strong optical confinement in hybrid plasmonic nanocavities formed by silver semi-cylindrical nanoshells (SCNS) and a planar gold film. The strong interaction between the Ag SCNS and the infinite Au film results in a hybrid plasmon, which splits into bonding and antibonding plasmon resonance modes. Such plano-concave nanocavities exhibit much improved confinement capability and relaxes the requirements on the spacer thickness. In addition, the hybrid resonance enables a large tuning range from the visible to the near-infrared by adjusting the modulation depth of the SCNSs. Such optical performances lay the basis for exploring applications in surface enhanced Raman spectroscopy, refractive-index sensors, plasmonic absorbers, notch filters, and ultrafast optical switching devices.

## Results and Discussion

### Plasmonic hybridization enhanced by nanocavity confinement

**Figure 1:**
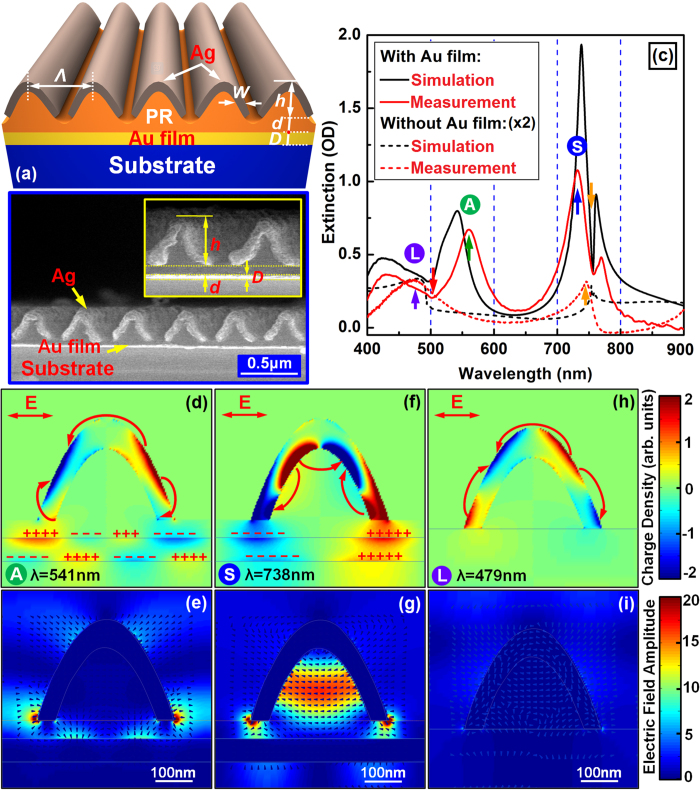
(**a**) 3D illustration of the arrays of the plano-semi-cylindrical-nanoshell nanocavities. (**b**) SEM image of cross-section profile of the plano-semi-cylindrical-nanoshell nanocavity arrays. (**c**) Measurement and calculation results of the reflective optical extinction spectra of the semi-cylindrical silver nanoshells (without gold film) and the plano-semi-cylindrical-nanoshell nanocavities (with gold film). (**d**), (**e**), (**f**): the calculated charge density distribution for the nanocavities at 541 and 738 nm, and for the pure nanoshells at 479 nm, respectively. (**g**), (**h**), (**i**): the calculated optical electric field distribution, corresponding to (**d**),(**e**), (**f**), respectively.

Figure 1(a) shows th e three-dimensional (3D) illustration of the periodic arrays of the plano-concave nanocavities formed by the semi-cylindrical-nanoshells and the infinite gold film. The design and fabrication of such structures are detailed in the methods section. Here the “nanocavity” effect refers to the enhanced confinement of optical electric field in the enclosed space by the SCNS and the gold film through plasmonic hybridization. The structural parameters of the nanocavities and the arrays are as defined in Fig. 1(a). Figure 1(b) shows the scanning electron microscopic (SEM) image of the cross-sectional profile of the nanocavity arrays and the inset of Fig. 1(b) presents an enlarged view of a local area. It is clearly shown that the oblique-deposited silver film discontinuously covers the photoresist (PR) grating lines, forming semi-cylindrical nanoshells extending infinitely along the PR nanolines. The SCNSs have an edge-to-edge separation of about *g* = 100 nm and a silver thickness of *t* = 80 nm. The SCNSs are separated from the Au film by a spacer thickness of *d* = 55 nm. Considering that the PR grating has a modulation depth of about *h* = 180 nm, the plano-concave nanocavity has a length of about *L* = 55+180 = 235 nm. The nanocavity arrays have a period of *Λ* = 500 nm. The planar gold film has a thickness of *D* = 45 nm.

Figure 1(c) shows measurement (red curves) and calculation (black curves) results of the reflective optical extinction spectra of the semi-cylindrical silver nanoshells (without underneath gold film, dashed curves) and the plano-semi-cylindrical nanocavities (with underneath gold film, solid curves). The solid red curve in Fig. 1(c) shows the measured reflective optical extinction spectrum at normal incidence for TM polarization (perpendicular to the extending direction of the semi-cylindrical nanoshells). Two obvious optical extinction peaks are observed with a relatively weaker one “A” at λ = 541 nm, as marked by an upward green arrow, and a stronger one “B” at λ = 738 nm, as marked by an upward blue arrow. Meanwhile, two dip features can be observed with the solid red curve, where a relatively broad one at 500 nm and a narrow one at 756 nm, as marked by a downward red and yellow arrow, respectively. Simulations using the same parameters as given in Fig. 1(a) show good agreement with the measurements, as shown in Fig. 1(c) by the black solid curve.

In addition, we also measured and calculated the spectroscopic response of pure SCNSs without the underneath gold film, as shown by the dashed red and black curves in Fig. 1(c), respectively. The same structural parameters were employed for the SCNSs as those in the nanocavity arrays. The calculations also show good agreement with the experiments. However, neither the strong-peak nor the narrow-dip features in the solid curves can be observed in the dashed ones, implying that they are unique performances of the nanocavities due to the enhancement by the gold film. Instead, two extremely weak peaks (amplitude doubled in the figure) can be observed with a broad one “L” at about 479 nm and a narrow one at about 750 nm, as marked by an upward purple and yellow arrow, respectively.

For understanding the photophysical nature of the spectroscopic performances in Fig. 1(c), we calculated the charge density distribution in the plane of incidence with the light polarized perpendicular to the nanoshells (TM polarization). Figure 1(d), (e) and (f) show the calculation results at 541 nm (peak “A” in the solid curves), 738 nm (peak “B” in the solid curves), and 479 nm (peak “L” in the dashed curves), respectively. Furthermore, we calculated the corresponding optical electric field distribution, as shown in Fig. 1(g), (h) and (i), respectively. In the near-field mapping in Fig. 1(g)–(i), the colors represent the magnitude of the electric field and the arrows indicate directions of the excited electric fields.

For pure semi-cylindrical nanoshells without the gold film, we observed tri-dipole resonance^29^,^30^,^31^,^32^,^33^,^34^,^35^,^36^,^37^ at λ = 479 nm, as shown in Fig. 1(f), and indicated by the three curved arrows. The charges are distributed mainly on the outer surface of the nanoshells, producing weak radiations into the free space, as shown in Fig. 1(i). However, when an infinite gold film is inserted underneath the SCNSs facing the concaved side of the SCNSs to form nanocavities, strong interaction between the SCNSs and the Au film was induced, so that both the charge-density and near-field distribution are strongly modulated in the SCNSs and the Au film. This introduced two different hybrid plasmons, which correspond to the resonance “A” at 541 nm and “B” at 738 nm in Fig. 1(c). For resonance “A” at 541 nm, high-density charges are distributed on the outer surface of the nanoshells, as shown in Fig. 1(d). However, due to the coupling with gold film, the space charge polarization was pulled toward to the feet of the SCNSs, as compared with the pure SCNSs in Fig. 1(f). In particular, different types of charges are accumulated on the outer and the inner edges of the SCNSs at the feet, which is defined as antibonding LSPR mode^22^,^23^,^26^. Such antibonding resonance mode is more convincingly verified by the asymmetric spatial-charge distribution across the thin metal film^33^,^34^,^35^, as marked by plus “+” and minus “−” charges in Fig. 1(d). Therefore, the extinction peak of “A” at 541 nm in Fig. 1(c) is caused by the hybridization between the two asymmetric modes, i.e. LSPR at the feet of SCNSs and SPP along the Au film. Figure 1(g) shows the distribution of the corresponding optical electric field, where relatively stronger field-enhancement is observed at the external corners at the feet of the SCNS than those in the spacer layer and into the Au film. Nevertheless, penetration into the gold film due to asymmetric SPP can be obviously observed in Fig. 1(g), which induces more optical extinction if compared with the symmetric resonance mode as shown in Fig. 1 (h).

For the resonance “B” at 738 nm, optical extinction as large as 1.15 OD has been measured, although this value is even smaller than the calculation results. Both the measurement and the simulation show that this resonance mode is not dependent on the angle of incidence, implying that it was not excited by grating-diffraction-related processes. Figure 1(f) illustrates the charge density distribution at the spectral position “B” in Fig. 1(c). The hybrid effects excite the symmetric SPP of the Au film, which corresponds to the symmetric spatial distribution across the thin metal film, as marked by the “+” and “−” charges. The symmetric SPP mode usually has small attenuation constant and is termed as long-range SPP^33^,^34^,^35^,^36^,^37^. Therefore, it induced much stronger coupling with LSPR to modulate significantly the charge distribution in the SCNSs, leading to much more strongly modulated optical electric field distribution in the hybrid system. The calculated charge density distribution in the SCNS in Fig. 1(e) shows clear antiphase charge-oscillation, as compared to Fig. 1(d), those for the resonance modes featured by spectral peaks “A” and “L”. This defines a bonding mode by the interaction between two symmetric resonances: the LSPR in the SCNS and the SPR in the gold film. As shown in Fig. 1(e), this resonance mode concentrates the majority of charges on the inner surface of the SCNS. Thus, the strong polarization is confined in the SCNS, which couples with the symmetric SPP, inducing significantly enhanced electric field inside the nanocavity formed by the SCNS and the gold film, as shown in Fig. 1(h). The vectors of the charge-accumulation-induced electric field in the SCNS are parallel to the surface of gold film, indicating formation of in-plane resonance inside the enclosed plasmonic nanocavity. In the nanocavities, the highest intensity of the electric field is 10 times higher than that inside a pure SCNS without the planar gold film. This confirms strong confinement of optical electric field inside such plano-semi-cylindrical nanocavities.

Furthermore, two optical extinction dips can be observed in Fig. 1(c), as marked by the red and yellow arrows, which are highly sensitive to the angle of incidence. The angle resolved tuning properties of them are demonstrated in Fig. S1(a) in the supporting information. We can attribute the relatively shallow dip at about 500 nm to the Fano coupling between the LSPR “A” and the Rayleigh anomaly (RA) of the grating diffraction^38^,^39^, which is determined by:


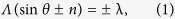


where λ is the RA wavelength, *Λ* is the grating period, and *θ* is the angle of incidence, *n* is the refractive index of the medium supporting the propagation of the anomaly diffraction. For *θ* = 0, *Λ* = 500 nm, *n* = 1 (in free space), we have exactly λ = 500 nm, which agrees well with the observation in Fig. S1(a). However, the relatively deep and narrow dip at 756 nm can be specified as the coupling between the propagating SPP at the Au/substrate interface and the hybrid plasmon designated as “B”, which exhibits Fano lineshape in the spectrum in Fig. 1(c). Such coupling effect has been verified by previous publications^5^,^6^,^7^,^8^. and can be observed in our simulation results of field distribution in Fig. S1(b).

Further important features in Fig. 1(c) that need to be clarified lie in the spectroscopic response of the pure SCNSs arrays without the underneath Au film. In the comparison between the solid and dashed curves, the very weak “dashed peaks” have excellent correspondence with the strong “solid dips”. Fig. S2(a) shows that these peaks split into two modes when the incident angle was increased from 0 to 2°, implying that they are both diffraction-anomaly induced surface propagation mode. According to equation (1), one mode is propagating on the top surface of the grating with *n* = 1 and λ = 500 nm and the other on the bottom of the grating with n ≈ 1.5 and λ ≈ 750 nm for normal incidence. These rough evaluations not only agree well with the data in Fig. S2, but also explain the excitation of propagating SPR at the Au/substrate interface, which is located at the narrow dip at about 750 nm in the solid curves in Fig. 1(c).

### Tunability of the hybrid plasmons

#### Dependence on the length of the nanocavity

**Figure 2:**
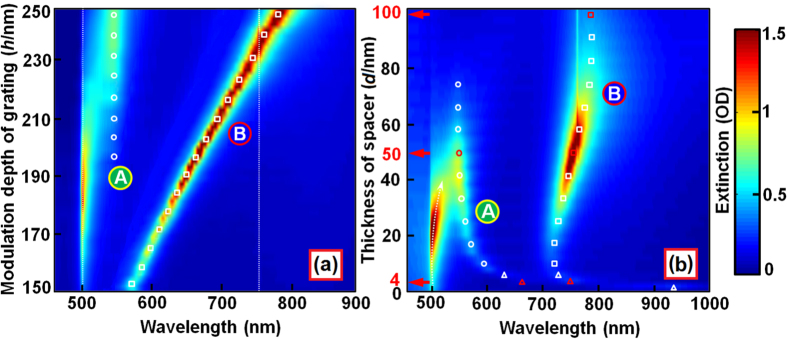
(**a**) and (**b**): 3-D spectroscopic response of the hybrid plasmons as a function of modulation depth of PR grating and thickness of the spacer layer, respectively.

Changing the length of the plano-semi-cylindrical nanoshell nanocavities can be achieved by changing the modulation depth of the template PR grating, which can be accomplished in the stage of interference lithography. However, changing the modulation depth of the precursor PR grating also changes the total circumference length of the Ag nanoshell in the cross-section perpendicular to the grating lines, thus, modifying the LSPR in the SCNS. All of these mechanisms will tune the hybrid resonance mode at “B” to the red with increasing the length of the nanocavities. This is confirmed by the simulation results in Fig. 2(a).

In the simulation, the thickness of Au film was set to 100 nm and other structural parameters were set to similar values as described in section 2.1. As shown in Fig. 2(a), when the modulation depth of the PR grating was increased from 150 to 250 nm, corresponding to an increase in the length of the nanocavities from 205 to 305 nm, the spectrum of the hybrid mode “B” shifts from about 750 to 785 nm, as indicated by the empty squares.

However, increasing the nanocavity length did not change either the thickness of the Ag nanoshells or that of the spacer layer. Thus, the antibonding mode of LSPR in both the SCNS and the Au film was not modified and the hybridization between them stayed fixed at about 550 nm, as shown in Fig. 2(a) by the empty circles. Meanwhile, the RA at about 500 nm shifts slightly to approach the resonance mode “A”.

#### Dependence on the thickness of the spacer layer

Figure 2(b) plots the calculated three-dimensional distribution of the reflective optical extinction as a function of wavelength and spacer-layer thickness (*d*), where the modulation depth of the SCNSs was fixed at *h* = 260 nm and the thickness of the space layer was increased from 0 to100 nm. Increasing the thickness of the spacer layer not only reduces the coupling strength between the SCNSs and the Au film, but also modifies the environment of the propagation SPP at the interface between the Au film and the spacer medium. For *d* < 6 nm, the hybrid plasmons “A” and “B” are nearly overlapped and cannot be resolved completely by their spectral features, as marked by empty triangles, and both exhibit a distinct blue shift as the space layer increases. This is based on the quickly reduced near-field interaction between SCNSs and the Au film^5^,^9^,^10^,^11^,^12^,^13^,^14^. However, for *d* > 6 nm, the hybrid plasmons split into “B” and “A”, which shift to opposite directions with “B” to the red and “A” to the blue, as the thickness of the spacer layer was increased further, as shown in Fig. 2(b). To understand the photophysical mechanisms for the tuning dynamics by changing the spacer-layer thickness, we calculated the plasmonic charge density distribution at some typical excitation wavelengths and show the results in Fig. S3. Related discussions are included in the second part of the Supporting Information.

#### Dependence on the angle of incidence

**Figure 3:**
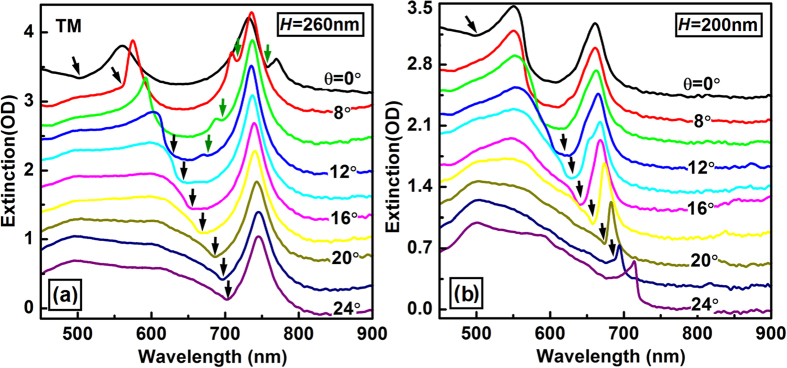
Measurements on the angle-resolved tuning properties of the optical extinction spectra of the hybrid plasmons with the SCNS’s modulation depth set to (**a**) *H* = 260 nm and (b) *H* = 200 nm.

To verify above proposed mechanisms and to characterize the dependence of the spectroscopic response of the plano-semi-cylindrical nanocavities on the angle of incidence, we performed measurements on the angle-resolved tuning properties of the reflective optical extinction spectrum, as shown in Fig. 3. Figure 3(a) show the measurement results on two samples with *h* = 260 and 200 nm, respectively. Other structural parameters of these samples are the same as those in Fig. 1(b). For *h* = 260 nm, resonance of two hybrid plasmons can be observed at 550 and 750 nm at normal incidence, corresponding to the anti-bonding and bonding modes, respectively, as shown in Fig. 3(a). As the incident angle was increased from 0 to 24 degrees, the anti-bonding mode shifts slightly the red and became merged with the broad-band background. However, the bonding mode stayed almost fixed with its peak wavelength located at about 750 nm. Meanwhile, the RA shifted from about 500 to about 710 nm, which was coupled with the anti-bonding mode at smaller *θ* and modified the bonding mode at larger *θ*, as indicated by the black arrows. Since the RA and the antibonding mode have comparable strength, the slight red shift of the antibonding mode resulted mainly from its interaction with the red-shifted RA, as indicated by the downward black arrows in Fig. 3(a). The bonding mode has a rough bandwidth of 30–40 nm at FWHM, which was reduced due to the modification by the diffraction anomaly at larger angle of incidence. Furthermore, Fano-like coupling between the bonding mode and the -1 order of the propagating SPR shifts to the blue with increasing the angle of incidence, as indicated by the downward green arrows in Fig. 3(b). The peak optical extinction of the bonding mode is as large as 1.5 OD, corresponding to a 97% modulation on the reflection of light.

As the value of *h* was reduced to 200 nm, which corresponds to the reduction in length of the nanocavity or the modulation depth of the grating, the antibonding modes stayed unchanged with its spectrum still peaked at about 550 nm. However, the resonance spectrum of the bonding mode shifted to a shorter wavelength of about 660 nm at normal incidence due to the reduction of *h*, as shown in Fig. 3(b). The bonding mode is a resonance of the nanocavities, the reduction in the cavity length and in the size of the SCNS led to blue-shift of the resonance mode. As the incident angle increasing from 8 to 24 degrees, stronger Fano-like couplings between the RA diffraction and the bonding plasmon can be observed, which are marked by black arrows in Fig. 3(b). Since the bonding plasmon stays fixed and the RA shifts to the red, the Fano-like coupling led to a narrowing effect in the resonance spectrum of the bonding mode, as can be observed clearly in Fig. 3(b). Efficient optical extinction can still be observed with the bonding resonance mode in the plasmonic nanocavities.

In conclusion, plasmonic hybridization in nanocavities consisting of silver semi-cylindrical nanoshells and a planar Au film was investigated. Localized anti-bonding and bonding plasmonic resonance modes were excited in the semi-cylindrical nanoshells, which induced asymmetric and symmetric surface plasmon resonance in the continuous Au film, respectively. Interaction between the Ag SCNS and the Au film led to anti-bonding and bonding hybridization between the LSPR and propagating SPP within two different spectra with high efficiency. These two hybrid plasmons can be tuned in spectroscopic response by changing the spacer thickness, the nanocavity length, and angle of incidence. Optical reflection modulation as high as 97% has been achieved within a bandwidth of 30–40 nm in the visible to near infrared spectral range. The high-efficiency and highly tunable hybrid plasmons presented in this work are important for exploring high-sensitivity biosensors, high-efficiency optical notch filters, and ultrafast optical switching devices.

## Methods

### Design and fabrication of the structures

Gold was deposited on a well cleaned silica substrate using thermal evaporation at a speed of about 0.2 nm/sec to produce a continuous film as thick as 45 nm. A photoresist (PR) grating with a period of 500 nm was fabricated using interference lithography on the above finished gold film, where a He-Cd laser at 325 nm was used as the UV light source and S1805 positive photoresist was used to record the grating. The remaining photoresist between the grating and the gold film was defined as the spacer layer. Through controlling the exposure and development processes, we can effectively adjust the modulation depth and duty cycle of the grating and the spacer thickness. In the final stage, silver was obliquely evaporated onto the surface of the grating, so that disconnected nanoshells formed on the top session of the grating line. The grating was tilted by an angle of *α* ≈ tan^−1^(*Λ*/*h*), with respect to the horizontal plane, for the oblique deposition on both sides, where *Λ* and *h* are the period and modulation depth of the grating, respectively.

### Optical spectroscopic measurement

In the reflective optical extinction measurements, a halogen lamp (HL-2000) was used as the white light source. A USB4000 fiber spectrometer from Ocean Optics was used to measure the reflection spectrum, which has a resolution of 2 nm and an effective spectral band from 340 to 1000 nm. The samples were mounted on a rotation stage with the angular resolution of 1 degree, so that the angle-resolved tuning properties can be characterized. For measurements at normal incidence, the reflected beam was collected by the fiber spectrometer after being reflected by a glass plate inserted between the sample and the white-light source. The optical extinction spectrum was calculated by −log_10_[I(λ)/I_0_(λ)], where I(λ) is the reflection spectrum by the sample and I_0_(λ) is that by a 45-nm Au film arranged in the same geometry.

### Numerical simulations

The simulation on the reflective optical extinction spectra, the electronic field distribution, the charge density distribution has been performed using FDTD (FDTD solution, Lumerical, Vancouver, Canada). The simulation was based on the bloch boundary conditions along the direction perpendicular to the grating lines with each unit cell containing a semi-cylindrical nanoshell and a segment of the continuous gold film. Perfectly matched layers were assumed along the normal to the grating plane. A mesh size as fine as 2 nm was employed. The incident plane wave was assumed to have a transverse-magnetic polarization, the optical constants for gold and silver were adapted from ref. 40 and the whole structures were assumed to be located in a homogeneous medium with a refractive index of *n* = 1.

## Additional Information

**How to cite this article**: Liu, F. *et al*. Plasmonic plano-semi-cylindrical nanocavities with high-efficiency local-field confinement. *Sci. Rep.* **7**, 40071; doi: 10.1038/srep40071 (2017).

**Publisher's note:** Springer Nature remains neutral with regard to jurisdictional claims in published maps and institutional affiliations.

## Supplementary Material

Supplementary Information
