# Supplementary material for: Plasmonic plano-semi-cylindrical nanocavities with high-efficiency local-field confinement
Source: Sci Rep. 2017 Jan 11;7:40071. doi: 10.1038/srep40071 (PMC5225429; doi:10.1038/srep40071)
Supplement: Supplementary Information [file srep40071-s1.doc]

**Supplementary Information**

**Plasmonic plano-semi-cylindrical nanocavities with high-efficiency local-field confinement**

Feifei Liu, Xinping Zhang,* and Xiaohui Fang

*Institute of Information Photonics Technology and College of Applied Sciences, Beijing University of Technology, Beijing 100124, P. R. China*

***Email: zhangxinping@bjut.edu.cn

**The calculated angle-resolved tuning properties of SCNS with and without Au film.** Figure S1 (a) shows the calculated reflective extinction spectra of the plano-concave nanocavities at normal incidence and at an oblique incident with θ=2°. Figure S1 (b) shows the near-field distribution at λ=756 nm for the normal incident.

Figure S2 shows the spectroscopic response of pure semi-cylindrical-nanoshells (SCNSs) without the underneath Au film at normal incidence and at an oblique incidence with θ=2°.


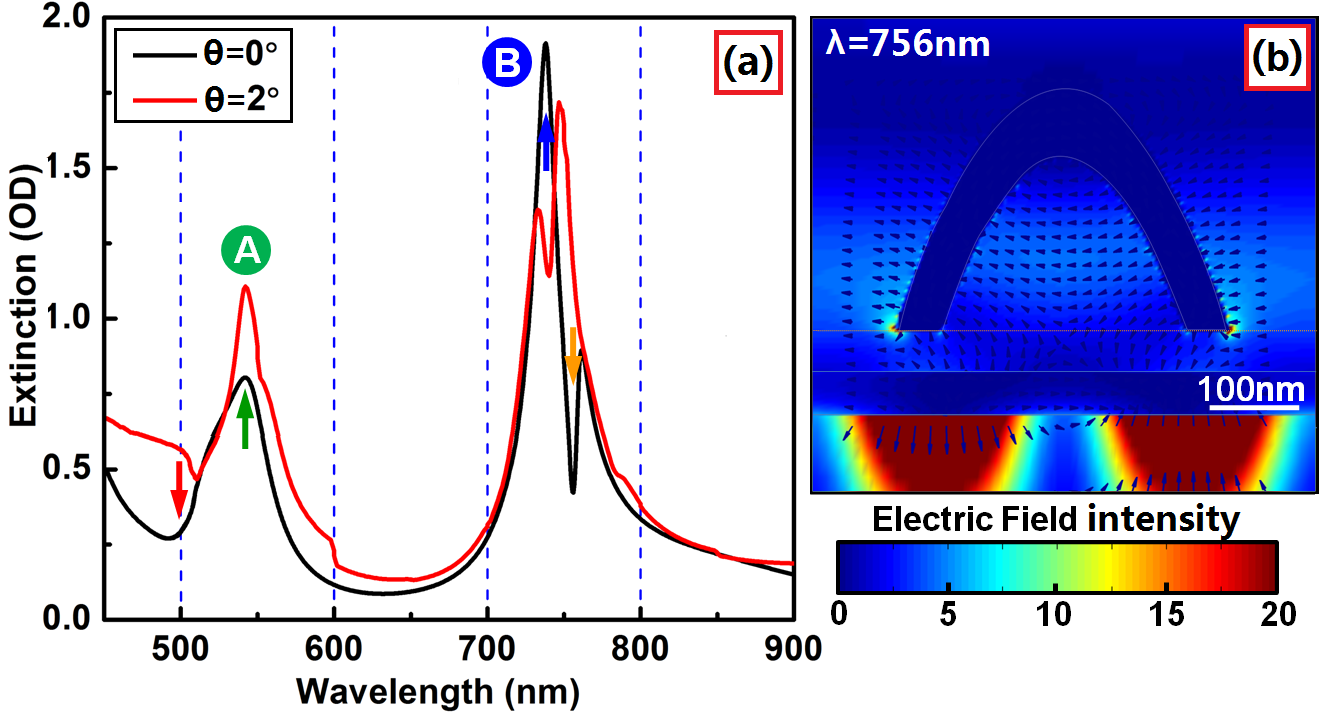


**Figure S1.** (a) Reflective optical extinction spectroscopic response of the hybrid plasmons at incident angles of θ=0° (black curve) and θ=2° (red curve). (b) The near-field distribution at λ=756 nm for normal incidence (*θ*=0°).


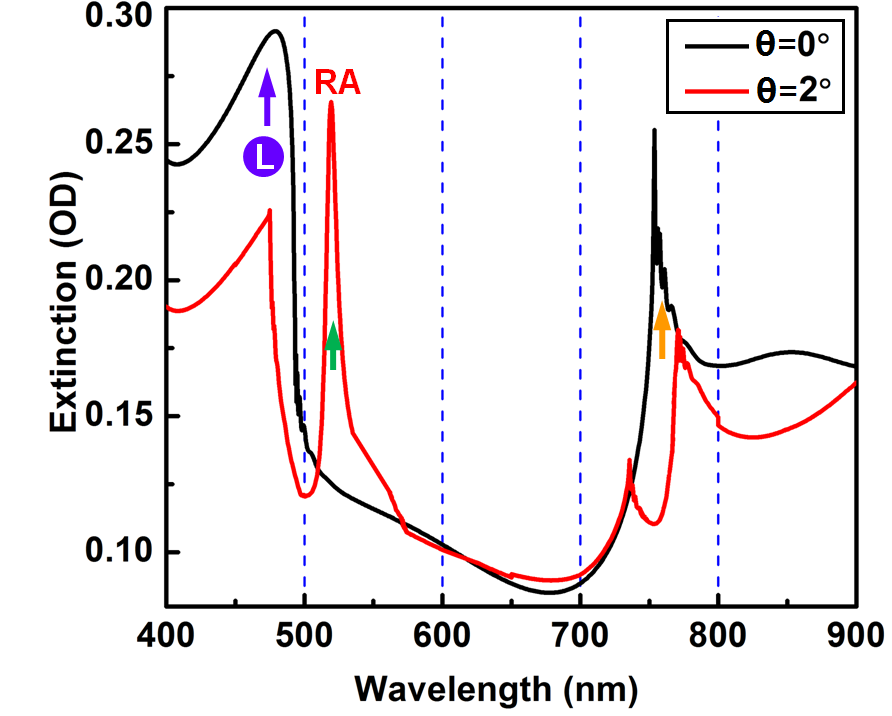


**Figure S2.** The calculated reflective optical extinction spectroscopic response of pure SCNSs without the Au film at incident angles of *θ*=0° (black curve) and *θ*=2° (red curve).

**The photophysical mechanisms for the tuning dynamics by changing the Spacer-layer thickness.** Fig. S3 shows the charge density distribution at typical spectral positions, where the thickness of separate spacer is *d*=4, 50 and 100 nm, respectively. For *d*=4 nm, Fig. S3(a) show the reflective optical extinction spectrum with the resonance “A” and “B” located at 662 and 746 nm, respectively. Figs. S3(b) and S3(c) show the charge density distribution at 662 and 746 nm, respectively. The resonance at “A” and “B” are partially overlapped in spectrum. Therefore, the charge density distribution for “A” is to much extent influenced by that for “B”, since “B” mode is much stronger than “A”, as shown in Fig. S3(a). Comparison between Figs. S3(b) and S3(c) indicates that these two modes show similar (indicated by solid arrows) and different (indicated by dotted arrows) features. The strong coupling between the SCNS and the Au film results in the splitting of the hybrid plasmon into two resonances: the lower energy bonding mode “B” and the higher energy antibonding mode “A”. However, at a small spacer thickness, they are overlapped in spectrum and share some common response properties.

For *d*=50 nm, the “B” mode reaches its peak intensity of reflective optical extinction at about 750 nm and the “A” mode at about 540 nm separates sufficiently from “B” with relatively reduced strength in comparison with its peak intensity at *d*=40 nm (dashed spectrum), as shown in Fig. S3(d). Figs. S3(e) and S3(f) show the charge density distribution at 540 and 750 nm, corresponding to resonance modes “A” and “B”, respectively. Well-defined anti-bonding and bonding mode can be observed in both the SCNS and the Au film for resonance “A” and “B”, respectively, as indicated by the curved arrows in Figs. S3(e) and S3(f). Strong coupling between the SCNS and the Au film played main roles in these two hybrid modes. In particular, the nanocavity resonance made dominant contribution to the formation of mode “B”.

However, when the SCNSs are separated so largely from the Au film, so that the value of *d* reaches 100 nm, the strength of the hybridization between LSPR in the SCNS and the SPP in the Au film becomes much reduced. Although mode “A” is supposed to be observed at about 540 nm, as indicated by the upward arrow, it is absent in the spectrum or it is so weak that it was overwhelmed by the background optical extinction. These changes can be observed in Fig. S3(g) and the corresponding charge-density distributions are shown in Figs. S3(h) and S3(i), respectively. As shown in Fig. S3(h), the anti-bonding modes become much weak in both the SCNS and the Au film, in particular, the much increased spacer thickness reduced significantly the hybridization strength between them. The curved arrows depict the anti-bonding modes in the SCNS, which correspond to the intrinsic LSPR in the either leg of the nanoshell or the anti-bonding resonance modes in such a nanoshell. Therefore, the resonance mode “A” shows a continuous blue shifting as the spacer thickness increasing. Meanwhile, the reduction in the hybridization strength due to the increase in the spacer thickness can also be observed with the mode “B”, where the intensity of the optical extinction is reduced to less than 20% in Fig. S3(g) (0.56 OD) as compared with that in Fig. S3(d) (~3 OD). In particular, superimposition between the symmetric LSPR in the SCNS (the broadband spectrum peaked at 780 nm) and the propagating SPP in the Au film (the sharp peak at about 770 nm) without Fano coupling can be observed in Fig. S3(g). This confirms again that the increased spacer thickness has reduced significantly the hybridization strength between the two resonance modes in the SCNS and in the Au film. Therefore, the theoretical results in Fig. S3 verified convincingly that the resonance modes “A” and “B” are hybrid plasmons due to the interaction between the LSPR in the SCNS and the SPP in the Au film.

Additionally, the increase in the spacer thickness also increases the length of the nanocavity, leading to the redshift of the resonance mode “B”, which can be observed by comparing the resonance spectrum of mode “B” in Figs. S3(a), S3(d), and S3(c). Furthermore, the increase in the spacer thickness also increased the equivalent refractive index slightly above the Au film, which may also lead to the redshift of the mode “B”. If we look back at the results in Fig. 2(b), both of the reflective optical extinction modes at resonance “A” and “B” are weak for *d*>70 nm, which can be attributed to the reduced hybridization due to the increased separation between the SCNS and the Au film. Furthermore, much reduced response in both mode “A” and “B” can also be observed for *d*<20 nm, as can be observed in Fig. 2(b). This implies that a minimum separation distance between the SCNS and the Au film is required for achieving hybridized plasmons.


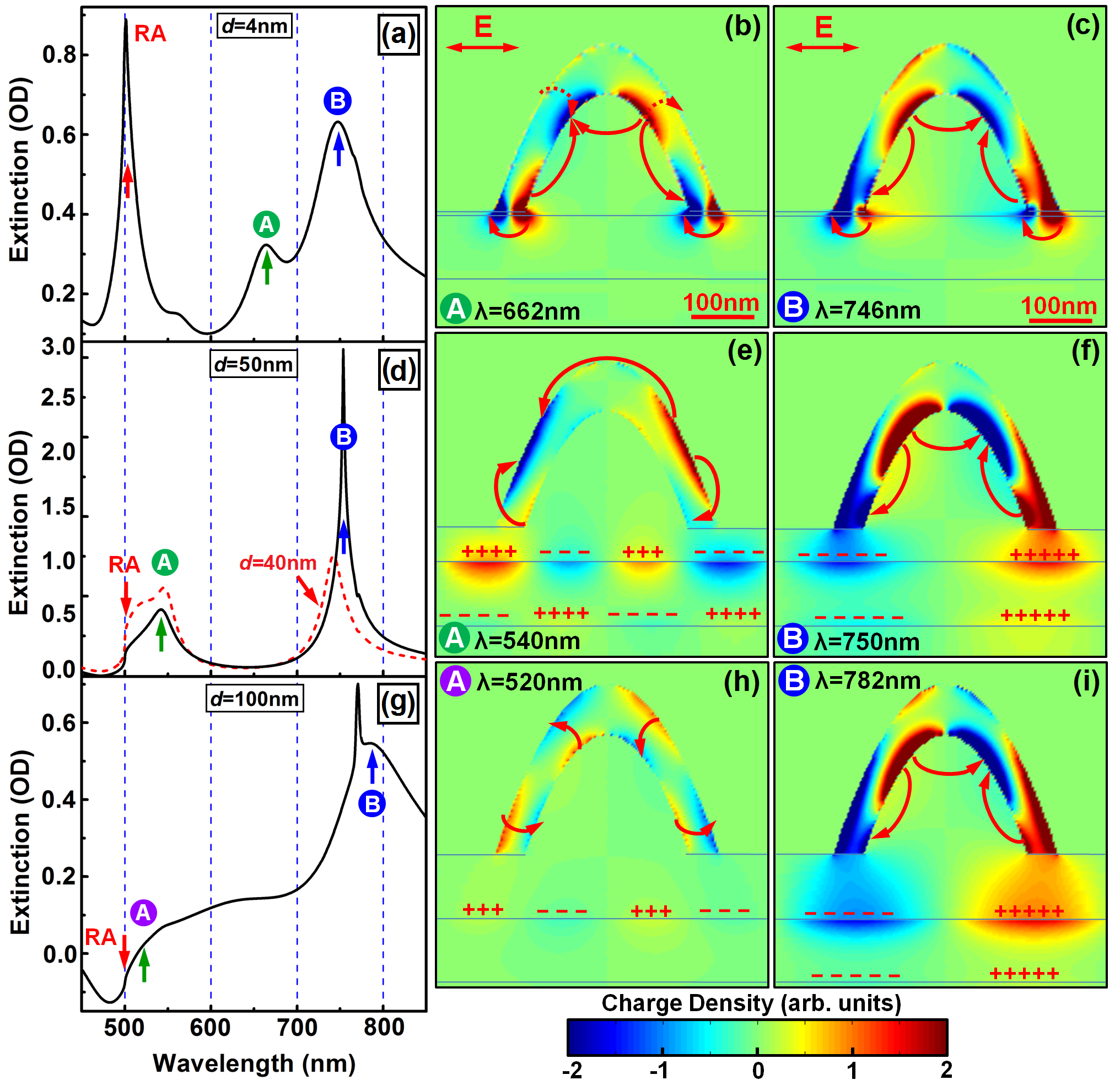


**Figure S3.** (a), (d) and (g): Spectroscopic response of the hybrid plasmons with the thickness of space layer changed from *d*=4, 50 nm to 100 nm. (b)-(c), (e)-(f), and (h)-(i): the corresponding charge density distribution at respective spectral positions of resonance modes “A” and “B”.
